# Multiple applications of flurbiprofen and chlorhexidine chips in patients with chronic periodontitis: a randomized, double blind, parallel, 2-arms clinical trial

**DOI:** 10.1111/j.1600-051X.2011.01779.x

**Published:** 2011-09-15

**Authors:** Eli E Machtei, Ilan Hirsh, Maher Falah, Eyal Shoshani, Avi Avramoff, Adel Penhasi

**Affiliations:** 1 Department of Periodontology, School of Graduate Dentistry, Rambam Health Care Campus and the Faculty of Medicine – Technion (I.I.T.)Haifa, Israel; 2 Dexcel PharmaOr Akiva, Israel

**Keywords:** anti-infective therapy, chronic periodontitis, controlled release device, NSAID, PerioChip

## Abstract

**Aim:**

The aim of the present randomized, double blind, parallel, 2-arm clinical study was to examine the safety and efficacy of frequent applications of chlorhexidine chip (CHX) and flurbiprofen chip (FBP) in patients with chronic periodontitis.

**Methods:**

Sixty patients were randomized into CHX and FBP groups. Following OHI and scaling and root planing (SRP), baseline pocket depth (PD) measurements, gingival recession and bleeding on probing (BOP) were performed and repeated at week 4 and 8. The assigned chip was placed at weeks 0, 1, 2, 3, 5, 7.

**Results:**

Mean PD reduction in the CHX group was 2.08 mm (7.17 to 5.09, *p* < 0.0001). Mean PD reduction in the FBP group was 2.27 mm (6.72 to 4.45, *p* < 0.0001). Ninety-seven percentage and 95% of these sites exhibited PD reduction ≥1 mm, while 38% and 34% of the sites exhibited PD ≥3 mm (FBP and CHX, respectively). Clinical attachment level gain (1.66 and 1.95 mm, respectively) was statistically significant (*p* < 0.0001). Baseline BOP dropped from 98% and 100% to 24% and 30% for the CHX and FBP groups, respectively (*p* < 0.0001).

**Conclusion:**

Frequent applications of CHX and FBP chips resulted in a significant improvement in the periodontal condition in these sites. Furthermore studies will be required to compare this new treatment regimen to SRP or SRP with single chip application.

Local delivery of antimicrobial agents as an adjunctive tool in the treatment of periodontal disease has been in use for over three decades now (Lindhe et al. 1979). An array of agents has been tested with varying degree of success. These are generally categorized into antibiotics, anti-bacterial agents and drugs modulating the inflammatory response.

Several antibiotics have been tested: Goodson et al. (1979) have shown that tetracycline (Tc)-filled hollow fibres placed in gingival pockets had a beneficial effect on both periodontal pockets and sub-gingival bacterial flora. Tetracycline was also loaded onto polymer strips and showed superior results for pocket reduction and bleeding on probing (BOP) compared to scaling and root planing (SRP) alone (Friesen et al. 2002). Other biodegradable carriers containing Tc as the active ingredient were also shown to be useful in the treatment of chronic periodontitis (Schwach-Abdellaoui et al. 2001, Liu et al. 2004). Doxycycline (Doxy) gel (10–14%) has also been studied for local delivery in periodontal pockets and shown to have good sub-gingival anti-microbial properties (Kim et al. 2002). Garrett et al. (1999) in a multi-centre study reported that local application of Doxy hyclate alone was as effective in pocket reduction and attachment gain as SRP. Likewise, minocycline HCl, another member in the Tc group, has shown to improve both periodontal parameters and to reduce perio-pathogenic flora when applied locally (Jones et al. 1994, Yeom et al.1997, McColl et al. 2006, Goodson et al. 2007). Nakagawa et al. (1991) using 2% minocycline-HCl ointment combined with SRP in patients with recurrent periodontal pockets have shown after 3 months, greater pocket reduction and elimination of perio-pathogenic microflora in these sites compared with sites treated with SRP alone. Several other antibiotics including amoxicillin with clavulanic acid (Abu Fanas et al. 1991), metronidazole (Noyan et al. 1997), azythromycin (Pradeep et al. 2008) and niridazole (Barat et al. 2006) were tested for local delivery in periodontal pockets with varying degrees of success.

Nonetheless, the use of low dose antibiotics in the periodontal pockets carries with it the risk of developing resistance, Kim et al. (2009) have concluded that local administration of Doxy can be identified in the systemic circulation at a level that has no antibiotic effect. Consequently, Larsen & Fiehn (1997) in an in vitro study and Rodrigues et al. (2004) in a human study, both reported the development of microbial resistance following the administration of metronidazole, minocycline and Tc.

Antibiotic agents with anti-microbial properties have also been tested for local delivery in periodontal disease. Of these, chlorhexidine gluconate (CHX) has been most intensively studied and been used clinically for two decades now (Heasman et al. 2001, Azmak et al. 2002). Recently, Paolantonio et al. (2008) concluded a clinical and microbiological a randomized multi-centre study on the effect of CHX chips (PerioChip®) which is a cross-linked biodegradable matrix of hydrolysed gelatin containing chlorhexidine gluconate 2.5 mg, combined with SRP, to SRP alone. Pockets depth (PD) reduction and clinical attachment level (CAL) gain were significantly greater 6 months after treatment in the combined treatment group. These findings are in agreement with previous findings of yet another multi-centre study (Soskolne et al. 1997). Other agents with anti-microbial properties have shown to have some effect on periodontal disease when applied sub-gingivaly. These include sanguinarine (Polson et al. 1996), silver ions (Straub et al. 2001), hyalurinan (Johannsen et al. 2009), chitosan (Wang et al. 2009), superoxide (Petelin et al. 2000) and even herbal medication (Hirasawa et al. 2002, Sastravaha et al. 2003).

Finally, drugs which modulate the host inflammatory response were also tested in both systemic and local application for the treatment of chronic periodontitis (Cetin et al. 2005). Tonetti & Chapple (2011) have recently concluded that non-steroidal anti-inflammatory drugs (NSAID) such as FBP may alter the course of periodontal disease in both animal model and human.

Nonetheless, the use of these control release devises (CRD) had its limitations: Radvar et al. (1996) in a comparative study of three CRD-containing antibiotics reported that while pocket depth (PD) reduction was slightly greater in the SRP + CRD groups, CAL gain was not statistically significant compared to SRP alone. Pavia et al. (2004) in a meta-analysis of the effectiveness of SRP with CRD containing metronidazole compared to SRP alone showed significant but small (0.2 mm) greater CAL gain for the combined treatment.

Thus, the purpose of the present randomized, double blind, parallel, 2-arm clinical study was to examine the safety and efficacy of frequent application of PerioChip® and Flurbiprofen Chip® (FBP group) in patients with chronic periodontitis.

## Material and methods

The study was initially approved by the institutional review board of the Rambam health care campus. Patients seeking periodontal treatment at the RHCC School of Graduate Dentistry were screened for this study [First patient enrolment-4th August 2009; Last patient follow-up (last visit)-8th June 2010]. To be eligible for the study, the following inclusion criteria were employed: chronic periodontitis with at least two teeth with periodontal pockets of 5–9 mm in depth (potential target teeth); demonstrating BOP in at least one site; radiographic evidence of alveolar bone loss. Patients were excluded for the following reasons: (i) tooth-related pathology that might be associated with the periodontal pocket; (ii) presence of three or more adjacent periodontal pockets on the same potential target tooth; (iii) systemic antibiotic therapy or use of NSAIDs prior to study entry (6 and 2 weeks, respectively) and throughout the study duration; (iv) usage of medication known to potentially result in gingival overgrowth; (v) type I diabetes or non-stable type II; (vi) known allergies to CHX or NSAID.

Eligible patients received a detailed explanation on the nature of the study and the alternatives, after which they signed an informed consent form. Next, full-mouth SRP was performed together with oral hygiene instructions. One to two weeks later, baseline measurements were obtained and the patients were randomized using predetermined computer-generated randomization scheme to receive either the PerioChip® (CHX group) or the FBP group. Randomized allocation sequence was done by using pre-determined computer-generated randomization scheme (SAS® version 9.2) to receive balanced random allocation of patients into the two study treatment. Only eligible patients at baseline visit were assigned with a sequenced randomization number. Each randomization number was randomly assigned to one of the two treatments FBP chip or CHX chip. The identity of the two treatments was kept blinded from the investigator and from the patient (double blind randomized trial design). In addition, one set of envelopes was provided to the investigator containing individual randomization codes; in event of an emergency, an unblinding breaking procedure was implemented. The randomized allocation sequence was generated by a statistician. The investigator was responsible for the patients’ enrolment. The patients were assigned randomly to one of the two blinde study treatments by the investigator.

Chips were inserted sub-gingivally as per the manufacturer's instruction for use. At weeks 1, 2, 3, 5 and 7 the same type of chip was re-inserted (in sites were insertion was met by resistance, PD was performed and if <5 mm, the chip was not inserted).

**Figure 1:**
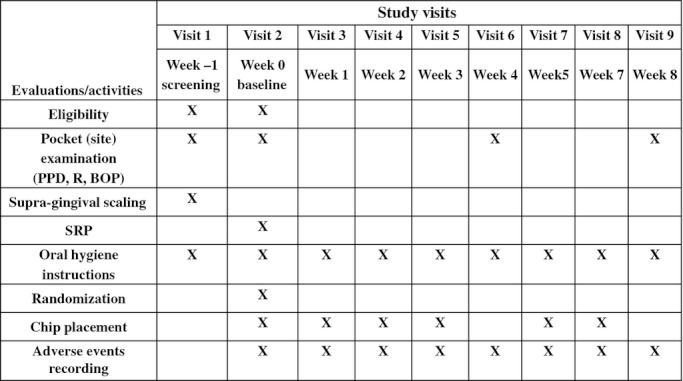
Study timeline.

Clinical measurements, which included PD, gingival recession (R) and BOP, were performed (using UNC probe; Hu-friedy, Chicago, IL, USA) at screening 0, 4 and 8 weeks (flow chart, Fig. 1). Clinical attachment level was calculated as the sum of PD and R. PD reduction, 0–8 weeks was defined as the primary outcome variable, while CAL gain and changes in per cent sites with BOP 0–8 weeks were set as secondary outcome variables.

Patients were instructed to report of any adverse event (AE); furthermore, at each visit they were actively approached to inquire of any AE that they might have experienced.

A total of 60 patients were recruited into the study, 30 in each group. Sample size was determined using a power calculation analysis to detect significant changes in PD (baseline to 8 weeks), for each treatment modality. Of these, 28 were female patients and 32 male patients with slightly greater female: male ratio in the CHX group (68%) compared to 38% in the FBP group (*p* = 0.02, Fisher's exact test). One patient (CHX group) withdrew (attended only the baseline visit); consequently, 59 patients, 30 FBP and 29 CHX completed the study and are reported in the present article. Less than 10% of these patients were smokers: three in the FBP and two in the CHX group.

### Statistical methodology

For each chosen pocket in a patient, we measured repeatedly at three main time points: 0, 4 and 8 weeks, three main parameters, PPD, CAL, and BOP. PPD and CAL are continuous parameters assumed to be approximately normally distributed, and BOP is a binary parameter. The parameters (PPD, CAL and BOP) measured for a specific pocket and a specific time point were modelled by a generalized model (Nelder & Wedderburn 1972) with repeated covariate structure for specific patients (each correlated patient structure includes two different pockets with at least three time points).

To model the covariance structure in a patient the Generalized Estimating Equations (GEE) method proposed by Liang & Zeger (1986) was used.

For normal distributed variables (PPD, CAL) an identity link was used, for binary parameters (BOP, change of at least 1 mm, etc.) a logit link was used.

We used age and gender as confounding variables. Visit, preparation and the interaction between visit and preparation are the explanatory variables.

The subject was the randomization number, and was used for the repeated structure in the GEE equations.

Specific contrasts for the visit preparation were used to compute elected differences with 95% confidence intervals needed for our analysis. All treatment comparisons were two-sided at the 0.05 level of significance. The primary time-point for all analyses was 8 weeks.

Similar models were built for categorized variables such as success to achieve 1 or 2 mm reduction in PD.

## Results

**Figure 2:**
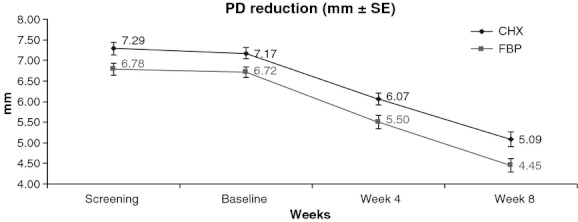
Primary endpoint – PD reduction.

Mean baseline PD was 7.17 ± 0.14 mm and 6.72 ± 0.13 mm for the CHX and FBP groups, respectively, which were very similar to the screening measurements for both groups (7.29 and 6.78 mm, respectively). Mean PD reduction (Fig. 2), from baseline to week 8 for CHX group was 2.08 ± 0.13 mm (7.17 to 5.09) which was statistically significant (*p* < 0.0001; GEE model). Mean PD reduction from baseline to week 8 visit for the FBP group was 2.27 ± 0.15 mm (6.72 to 4.45) which was also statistically different (*p* < 0.0001; GEE model). PD reduction 0–8 weeks was not different between groups (2.08 ± 0.13 mm for the CHX group and 2.2 ± 0.15 mm for the FBP groups; *p* > 0.05). When the extent of PD reduction was dichotomized, almost all these sites (95% and 97% for the CHX and FBP groups, respectively) had at least 1 mm reduction between baseline and 8 weeks (Table 1). More important, almost three quarters of these sites (72% and 73% for the CHX and FBP groups, respectively) had at least 2 mm reduction in PD and just over one-third of all these sites (34% and 38% for the CHX and FBP groups, respectively) had 3 mm or more pocket reduction in these sites. Small proportions of these pockets (7–17%) had a startling 4 mm. PD reduction during this period.

**Table 1:** Pocket depth reduction (dicatomized 1-4)

	At least 1 mm reduction		At least 2 mm reduction		At least 3 mm reduction		At least 4 mm reduction	
	BL-Wk.4	BL-Wk.8	BL-Wk.4	BL-Wk.8	BL-Wk.4	BL-Wk.8	BL-Wk.4	BL-Wk.8h
FBP	83%	(50/60)	97%	(58/60)	30%	(18/60)	73%	(44/60)	8%	(5/60)	38%	(23/60)	0%	(0/60)	17%	(10/60)
CHX	81%	(47/58)	95%	(55/58)	28%	(16/58)	72%	(42/58)	2%	(1/58)	34%	(20/58)	0%	(0/60)	7%	(4/58)

**Figure 3:**
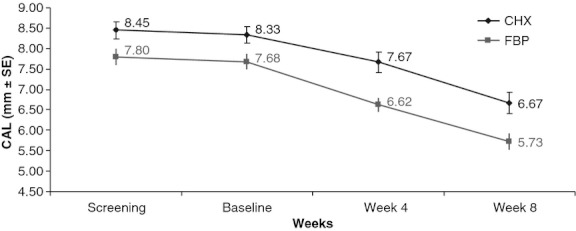
CAL reduction – baseline to 8 weeks.

Likewise, mean CAL reduction (Fig. 3) from baseline to week 8 for CHX group was 1.66 mm (8.33 to 6.67) which was statistically significant (*p* < 0.0001; GEE model). Mean CAL reduction from baseline to week 8 for FBP group was 1.95 mm (7.68 to 5.73) which was statistically significant (*p* < 0.0001; GEE model). Again, the differences between the groups were not statistically significant (*p* > 0.05).

**Figure 4:**
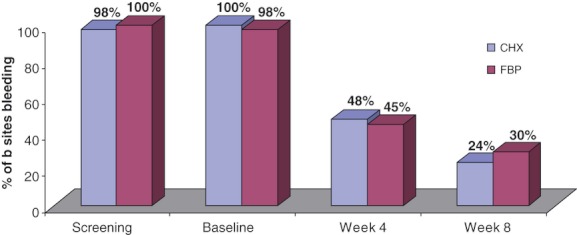
Changes in bleeding on probing.

Bleeding on probing was present, at baseline in all but few of these sites (100% and 98% for the CHX and FBP groups, respectively). These proportions dropped significantly at 4 weeks (48% and 45%) and further at week 8 (24% and 30%). Again, these proportions were not statistically different between the two groups in any of these time points (Fig. 4). The difference between treatments was not significant (*p* = 0.42, Fisher's Exact test).

On visit 7, 10 sites in the CHX group and 14 sites in the FBP group had PD <5 mm which precluded the placement of the chip. Likewise, on the final visit 14 and 19 sites, respectively, had shallow pockets.

Data were further sorted to compare treatment response between single *versus* multi-rooted teeth (Table 2). PD reduction in the single-rooted teeth (1.82 and 2.73 mm for the CHX and FBP groups, respectively) was not significantly different (GEE) compared to multi-rooted teeth (2.15 and 2.16 mm for the CHX and FBF groups, respectively). Likewise, CAL gain and BOP reduction between baseline and 8 weeks were similar.

**Table 2:** Changes in PD: multi-rooted *versus* single-rooted teeth

ΔPPD	FBP			CHX		
	Tooth type			Tooth type		
	Multi-root	Single-root	Other	Multi-root	Single-root	Other
Baseline/Screening	−0.09	0	0		−0.15	0.18	−0.5
Week 4/Baseline	−1.16	−1.55	−1		−1.15	−1.09	−0.83
Week 8/Baseline	−2.16	−2.73	−2.2		−2.15	−1.82	−2.17

Related AEs were minimal in both groups. These included mild gingival pain (2 FBP, 1 CHX), dentinal pain (1 FBP, 3 CHX), headache (0 FBP, 1 CHX) and gingivitis (0 FBP, 1 CHX). None of the patients in either group had experienced any severe adverse reaction to these chips.

## Discussion

Frequent application of CHX and FBP chips together with single session of SRP at baseline resulted in a mean PD reduction of more than 2 mm within 8 weeks in patients with chronic periodontitis.

Likewise, van Steenberghe et al. (1999) in a 1-year study of repeated minocycline application reported mean PD reduction of 1.9 mm in sites with initial PD >5 mm. Slightly smaller PD reduction (1.7 mm) was reported by Riep et al. (1999) using repeated metronidazole application in maintenance patients.

Mean CAL gain in the present study was 1.66–1.95 mm. These results exceed those of Friesen et al. (2002) study which reported mean CAL gain of 0.18 mm after 3 months when using repeated local application of Tc containing strips; while in other studies where repeated local delivery system (LDS) was used, CAL gains after 3 months were 1.1 mm using minocycline (van Steenberghe et al. 1999) and 1.31 mm using metronidazole (Riep et al. 1999). The greater CAL gain in the present study might be associated with the greater intensity of the application of the chips; this in turn results in higher local concentration of the drug that might be responsible for the greater effect. Bogren et al. (2008) using Doxy gel have shown that repeated annual application did not result any beneficial effect (neither clinically nor microbiologically) in maintenance patients with PD ≥5 mm.

Repeated LDS of antimicrobial agents is not necessarily limited to non-surgical therapy. Yoshinari et al. (2001) examined the effect of repeated minocycline application on the success of guided tissue regeneration using expanded polytetrafluoroethylene (ePTFE( membranes and reported significantly greater CAL gain (3.0 mm) compared to ePTFE alone (2.0 mm) in 1–3 wall intra-bony defects. Likewise, our group (Machtei et al. 2003) in a similar previous study of GTR in mandibular class II defects in smokers, reported greater CAL gain and bone regeneration in patients who had repeated metronidazole application as part of an aggressive anti-infective regiment.

Practically all sites had at least 1 mm. PD reduction at the end of the study, while 75% had ≥2 mm. PD reduction and over 1/3 of the sites exhibited PD reduction ≥3 mm. Thus, a great number of these sites initially targeted for surgical periodontal therapy, had improved to the extent that no further active therapy was needed. van Steenberghe et al. (1999) using similar protocol with minocycline reported ≥2 mm PD reduction in approximately 50% of the sites that were initially ≥5 mm.

The magnitude of improvement in PD and CAL in the present study (1.66–2.27 mm) was by far greater than what was reported in most studies for single application of LDS. However, the lack of SRP only control in the present study precludes any attempt to estimate the proportion of the adjunctive effect of the two chips. Single application of CHX chips (Heasman et al. 2001) resulted in mean PD of 0.55–0.78 mm after 3 and 6 months. To the contrary, Eickholz et al. (2002) reported greater PD reduction (3.1 mm) and similar CAL gain using SRP + single DOXY gel application, is 110 single rooted teeth. Pavia et al. (2003) in a meta-analysis of the efficacy of Tc (Tc fibres; Doxy and minocycline gel) in chronic periodontitis patients reported mean additional PD reduction and CAL gain of 0.6–0.8 mm and 0.33–0.74 mm for SRP + local Tc over SRP only.

Approximately 25% of these sites still had BOP at 8 weeks compared to 98–100% at baseline. This marked reduction in the proportions of sites that bled on probing represents significant reduction in inflammation and possibly bacterial load in these sites. Bogren et al. (2008) reported 37% of the sites to still exhibit BOP 3 months after SRP + LDS with Doxy; however, at baseline only 51% of these sites had BOP, thus the overall reduction in this study was merely 27% compared to 75% in our study. Likewise, McColl et al. (2006) in a 12 months randomized control study using 2% minocycline gel reported the drop in BOP to range between 0% and 50.5%. Goodson et al. (2007) have shown that LDS of antimicrobial drugs had a significant effect in reducing the red-complex bacteria in the periodontal pocket. The larger reduction in BOP observed in the present study is likely due to the anti-infective effect that the frequent application of these chips had on the microbial flora (CHX) and inflammatory response (FBP).

The CHX chips resulted in similar and even greater response compared to the FBP chips. This is the first study ever to compare these two agents which have such a different mode of action. Few studies are available where LDS containing NSAIDs were tested for the treatment of periodontal disease. Williams et al. (1988) using FBP gel that was applied daily into the gingival margins of beagle dogs, reported less bone loss and tooth loss compared to untreated animals. Li et al. (1996) using daily application of ketoprofen gel in rhesus monkeys reported alveolar bone gain compared to placebo control which exhibited net bone loss in an experimental periodontitis and spontaneous periodontitis model. This beneficial effect of the FBP reported in the present study was similar to that found for the CHX chips. Thus, further studies on drugs with local anti-inflammatory effect are warranted. These findings of a sizeable improvement, in both the CHX chip (with its anti-bacterial properties) and the FBP chips (with its anti-inflammatory properties), would tend to suggest that if both chips are used consecutively or simultaneously, it might result in even greater improvement in clinical parameters compared to when each of them is used individually; however, this hypothesis will need furthermore research to be substantiated.

In conclusion the frequent use of CHX or FBP chips in conjunction with single SRP visit, resulted in marked improvement in the periodontal condition in patients with chronic periodontitis. To further assess if this new mode of application is superior to single application or SRP only treatment, furthermore studies with such controls will be required.

## 

**Clinical relevance** *Scientific rationale for the study:* The use of LDS has shown some benefit in pocket reduction and resolution of inflammation. However, the extent of this improvement has been limited. CHX and to a lesser extent NSAID have shown beneficial effect in modulating periodontal inflammation when applied into the periodontal pocket. The aim of the present study was to explore the efficacy and safety of a new drug (FBP chip) and well-known LDS medication (CHX Chip) using a new, intensive placement protocol.

*Principal findings:* Mean PD reduction (2.08–2.27 mm), CAL gain (1.66–1.95 mm) and drop in BOP (68–76%) in the two groups was greater than previously reported with LDS using standard single application.

*Practical implications:* This new regimen for LDS application might replace the current single application mode to gain better improvement in periodontal parameters in patients with chronic periodontitis.
